# BTN3A2 Expression in Epithelial Ovarian Cancer Is Associated with Higher Tumor Infiltrating T Cells and a Better Prognosis

**DOI:** 10.1371/journal.pone.0038541

**Published:** 2012-06-07

**Authors:** Cécile Le Page, Alexandre Marineau, Patrick K. Bonza, Kurosh Rahimi, Louis Cyr, Ingrid Labouba, Jason Madore, Nathalie Delvoye, Anne-Marie Mes-Masson, Diane M. Provencher, Jean-François Cailhier

**Affiliations:** 1 Centre de Recherche du Centre Hospitalier de l’Université de Montréal (CRCHUM), Montreal, Quebec, Canada; 2 Institut du Cancer de Montréal, Montreal, Quebec, Canada; 3 Department of Pathology, Centre Hospitalier de l’Université de Montréal (CHUM), Montreal, Quebec, Canada; 4 Department of Medicine, Université de Montréal, Montreal, Quebec, Canada; 5 Division of Gynecologic-Oncology, Centre Hospitalier de l’Université de Montréal (CHUM), Montreal, Quebec, Canada; 6 Division of Nephrology, Centre Hospitalier de l’Université de Montréal (CHUM), Montreal, Quebec, Canada; Duke University Medical Center, United States of America

## Abstract

BTN3A2/BT3.2 butyrophilin mRNA expression by tumoral cells was previously identified as a prognostic factor in a small cohort of high grade serous epithelial ovarian cancer (HG-EOC). Here, we evaluated the prognostic value of BT3.2 at the protein level in specimen from 199 HG-EOC patients. As the only known role of butyrophilin proteins is in immune regulation, we evaluated the association between BT3.2 expression and intratumoral infiltration of immune cells by immunohistochemistry with specific antibodies against BT3.2, CD3, CD4, CD8, CD20, CD68 and CD206. Epithelial BT3.2 expression was significantly associated with longer overall survival and lower risk of disease progression (HR = 0.651, p = 0.006 and HR = 0.642, p = 0.002, respectively) and significantly associated with a higher density of infiltrating T cells, particularly CD4+ cells (0.272, p<0.001). We also observed a strong association between the relative density of CD206+ cells, as evaluated by the ratio of intratumoral CD206+/CD68+ expression, and risk of disease progression (HR = 1.355 p = 0.044, respectively). In conclusion, BT3.2 protein is a potential prognostic biomarker for the identification of HG-EOC patients with better outcome. In contrast, high CD206+/CD68+ expression is associated with high risk of disease progression. While the role of BT3.2 is still unknown, our result suggest that BT3.2 expression by epithelial cells may modulates the intratumoral infiltration of immune cells.

## Introduction

Epithelial ovarian cancer (EOC) is a leading cause of death among gynecologic malignancies [1]. Due to its lack of symptoms, this disease is often diagnosed at an advanced stage (stage III or IV) when the cancer has already spread to secondary sites. The standard treatment for these patients is surgery and platinum-based chemotherapy, although the disease often progresses even after surgery and becomes resistant to standard chemotherapy [2]. Consequently, the survival rate of patients with advanced-stage EOC is extremely low (<40%). Clinical variables, such as disease stage and residual disease, are prognostic [3], [4], but largely uninformative for those with advanced-stage disease.

In a previous study [5], we performed a mRNA expression profiling of high grade serous EOC tumor tissues to identify molecular markers of prognosis using an Affymetrix-based gene expression microarray platform. Among the prognostic markers identified, we found BTN3A2, also known as BT3.2 and BTF4, a gene belonging to the BT3 butyrophilin family, also a B7 subfamily. BTN3A2 mRNA was the most robust biomarker among 8 others tested. Not only did it have a prognostic significance in univariate and multivariate analyses, but it also showed the highest hazard ratio compared with the other molecular and clinical prognostic variables. High mRNA expression of BT3.2 was strongly associated with a good prognosis in relation to disease-free survival and overall survival. As an EOC prognostic marker it reached 87% specificity and 77% sensitivity to predict early recurrence (<18 months) in a cohort of 52 patients [5].

The butyrophilin (BTN) family contains BTN1A1, BTN2A1, BTN2A2, BTN2A3, BTN3A1, BTN3A2, BTN3A3, and BTN-Like members. Interestingly, these novel BTN family members share considerable homology with B7 family members, which have an IgV-like exoplasmic domain followed by another exoplasmic IgC-like domain. The cytoplasmic domain contains a protein binding SPRY/PRY B30.2 domain [6], [7], [8]. Unlike other BTN members, BT3.2 does not have a B30.2 domain in the intracellular region [7], [9]. BT3 molecules (BTN3A1/BT3.1, BTN3A2/BT3.2 and BTN3A3/BT3.3) are known to be expressed on endothelial cells [10], in the membrane layer surrounding milk fat-secreting droplet from mammary epithelial cells [11], on immune cells, and on some tumor cell lines [5], [12].

The B7 molecules can be negative or positive regulators of T cell activation. B7-H1/PD-L1, B7-H3, B7-H4, B7DC/PD-L2, B7S3, B7S1, BTNL2, BTN1A1, BTN2A2 can inhibit T cell proliferation delivering an inhibitory signal. Conversely, B7-H3 has been regarded as a positive and negative regulatory molecule able to stimulate or inhibit CD4+ and CD8+ T cells depending of the cellular context [13], [14], [15], [16]. Similarly, some BTN family members have recently emerged as new regulators of the immune system. These include BTNL2 [17]–[18] BT1.1, BT2.2 [19], BTNL1 [20], [21] and the BT3 molecules. For example, Yamashiro *et al.* [22] treated PMBC with an antibody against BT3.3 and observed an inhibition of CD4+ and CD8+ cell proliferation, as well as decreased cytokine secretion by both types of T cells. In another report, Cubillos-Ruiz *et al*. showed that BT3 expression on antigen-presenting cells (APC) inhibited *in vitro* T cell proliferation and Th1 cytokine secretion [23]. While these studies have looked at the function of B7 family members when expressed on the immune component, it is not clear how these molecules might affect immune response when expressed by other cell types. Indeed, published data supports the expression of BT3 on EOC cells in primary tissues and metastatic tissues [23]. In addition, Messal N. *et al*. recently reported that BT3 acts as a co-stimulatory molecule on CD4+ T cells and NK cells [24]. Interestingly, they observed a differential immuno-regulatory role of the different BT3 isoforms. Altogether this suggests that complex mechanisms may govern the function of the BT3 molecules. Further observations are needed to understand the exact role of these molecules in a context specific manner.

To better understand the role of BT3.2 in ovarian cancer and more particularly to confirm our observed association of mRNA BT3.2 expression with a good patient prognosis [5], we performed an immunohistochemistry analysis of BT3.2 protein expression on a large cohort of 199 high grade serous EOC patients and confirmed the association of BT3.2 and prognosis of EOC patients. In parallel, we analyzed the density of intratumoral immune cells and found a positive correlation between BT3.2 expression by EOC cells and intratumoral infiltration of T cells, whilst a high CD206+/CD68+ expression ratio was associated to shorter disease-free survival. These results suggest a role of BT3.2 in the tumor infiltration of immune cells.

## Materials and Methods

### Ethics Statement

Ethics approval was obtained by the local institutional ethics board (comité d’éthique de la recherche du Centre hospitalier de l’Université de Montréal). Tumor samples were collected and banked following appropriate written consent from patients undergoing surgery within the Oncology Department at the Centre Hospitalier de l’Université de Montréal from 1993 to 2010.

### Patients and Tissue Specimens

Tumor samples were collected and banked following appropriate consent from patients undergoing surgery within the Division of Gynecologic Oncology at the Centre Hospitalier de l'Université de Montréal from 1993 to 2010. An independent pathologist scored tumor grade and stage and a gynecologic oncologist scored tumor residual disease according to criteria from the International Federation of Gynecologists and Obstetricians [25]. Less than 10% of specimen were excluded on quality, and this was not correlated to age of sample. Clinical data on progression-free interval were defined according to scan imaging and level of blood CA125 [26]. Overall survival was defined as the time from surgery to death from ovarian cancer. Patients known to be still alive at time of analysis were censored at time of their last follow-up. Patient disease free survival (DFS) was calculated from the time of surgery until the first progression. Eligibility criteria for inclusion in the study were as follows: no pre-operative chemotherapeutic treatment for ovarian cancer, platinum-based post-operative chemotherapy treatment, high grade tumors, serous histopatholgy subtype and completed informed consent. All patients received a platinum-based chemotherapy as an initial therapy after surgery with the exeption of patients who died shortly (<3 months) after surgery. Patients who died from another disease were censored at time of last follow-up. A gynecologic oncologist reviewed the clinical data for all patients. For the disease-free progression study, only patients with clinical follow-up of at least 18 months or until disease recurrence were included. The characteristics of the tumors and patient outcome for the sample sets are summarized in Table 1.

**Table 1:** Clinical characteristics of the patients included in the cohort.

	Characteristics	Number of patients	Time (range)
**AGE**		199	61 yr (34–89 yr)
**STAGE**	I	10	
	II	20	
	III	140	
	IV	27	
**RES.DISEASE**	neg	23	
	>1 cm	26	
	1–2 cm	15	
	2 cm	61	
	milliary	15	
**SURVIVAL TIME**	Incidence of death	74	26 mo (1–74 mo)
**FOLLOW UP TIME**		118	40 mo (1–140 mo)
**DIS. PROGRESSION TIME**	<18 mo	82	9 mo (1–17 mo)
	>20 mo	93	50 mo (20–140 mo)
**BT3.2 staining**	<1	35	
	1–2	121	
	>2	42	
	missing	1	

Stage was divided in 4 categories according to FIGO classification. The histological grade of the tumor was evaluated by a pathologist and divided in two groups (grade 2 or 3) according to the FIGO grading system. Residual disease at surgery (Res. Disease) was evaluated in centimeter (cm) by a gyneco-oncologist. Neg =  no residual disease. Survival and follow up time is the time in months (mo) from the date of primary resection of the ovarian tumor until the event of death due to ovarian cancer or until the last contact with the patient. Disease progression time is the time of disease progression from the date of primary resection of the ovarian tumor until the first event of disease progression. BT3.2 staining is the staining intensity observed on the tissue microarray analysis with an antibody against BT3.2. Intensity 1: weak. Intensity 2: moderate. Intensity 3: strong. For additional details see Materials and Method section.

### Cell Cultures and Cell Pellets in Histogel

To verify the specifity of the BT3.2 antibodies, TOV-112D cells and derived cell lines were cultured in OSE medium (50∶50 mix of 199 and 105 Sigma mediums) supplemented with 0.5 µg/mL of amphotericin B, 50 µg/mL of gentamycin and 10% of FBS (fetal bovine serum). Derived cells media were also supplemented with 0.5 µg/mL of puromycin. Cells were maintained in an incubator humidified at 37°C with an atmosphere containing 5% of CO2 essentially as described [27]. Cells pellet embedded in paraffin were prepared as previously described [28].

### Plasmids

The BT3.1, BT3.2 and BT3.3 sequences were subcloned in the pef6v5 vectors and were a generous gift of Dr Rhodes (Cambridge, UK) [7]. All plasmids were verified by sequencing. The BT3 isoform sequences were PCR amplified and subcloned in the pENTR™/D-TOPO® (Invitrogen, Carlsbad, CA) and then recombined in the pLenti-CMV/TO-puoDest (Abgene) [29]. Lentiviral particles production and cell infection was done as previously reported [30].

### Tissue Microarray

Areas of tumor were selected based on review of a hematoxylin-eosin-stained slide. FFPE tumor blocks were then biopsied using a 0.6 mm diameter tissue arrayer and resultant cores were arrayed into a grid in a recipient paraffin block. The tissue array was composed of 260 ovarian cancer samples and 11 samples of areas from normal fallopian tubes of cancer patients. After review of clinical data 61 patients were excluded from the final analysis as they did not meet the inclusion criteria. This tissue array was then sectioned, stained with hematoxylin-eosin and received another pathology review to confirm tumor content.

### Immunohistochemistry (IHC)

Formalin fixed paraffin embedded tumors were sectioned at 4 µm and the slides were stained manualy on the bench or using the BenchMark XT automated stainer (Ventana Medical System Inc.). For the manual method, tissue sections were heated briefly at 50°C for 15 minutes, deparaffinized in toluene and rehydrated in an ethanol gradient. Slides were submerged in Tris-EDTA buffer (10 mM Tris base, 1 mM EDTA adjusted to pH 9.0) and pressure cooked for 15 min to unmask antigens. A 0.6 or 3% H_2_O_2_ treatment was used to eliminate endogenous peroxidase activity. The sections were blocked with a protein blocking serum-free reagent (DakoCytomation Inc., ON, Canada) and incubated with different antibodies for 60 or 120 min at room temperature. The optimal concentration for each primary antibody was determined by serial dilutions. Antibodies and IHC conditions are listed in Table S1. Tissues were incubated with a goat anti-mouse HRP-conjugated antibody (1∶150) (sc-2005, Santa Cruz Biotechnology, CA, USA). For BT3.2, tissues were incubated with a secondary biotinylated antibody (DakoCytomation Inc., ON, Canada) for 30 min followed by incubation with a streptavidin-peroxidase complex (DakoCytomation Inc., On, Canada) for 30 min at room temperature. Reaction products were developed using diaminobenzidine containing 0.3% H_2_O_2_ as a peroxidase substrate. Nuclei were counterstained with hematoxylin. With the automated stainer, antigen retrieval for CD206 and CD4 was carried out with Cell Conditioning 2 (VMSI; #950–123). Prediluted antibody was automatically dispensed, and the slides were incubated at 37°C for 30 min. UltraView DAB detection kit (VMSI; #760–091). Slides were counterstained with hematoxylin (VMSI; #760–2021). All sections were observed by light microscopy at 400× magnification. Substitution of the primary antibody with phosphate buffered saline served as a negative control.

### Immunofluxorescence (IF)

Formalin fixed paraffin embedded tumors were stained manualy after antigen retrieval in Tris-EDTA (10 mM Tris base, 1mM EDA adjusted to pH 9.0) for 15 min in pressur cooker. The sections were blocked with a protein blocking serum-free reagent (DakoCytomation Inc., ON, Canada) and incubated with the anti-CD68 antibodies for 90 min at room temperature. Tissues were then incubated with a goat anti-mouse Cy5 (1/200) for 45 min. Mouse IgG antibodies were then blocked overnight with M.O.M.™reagent (Vector Laboratories, Burlingame, CA). Anti-CD206 antibodies were incubated for 90 min at room temperature. Tissues were then incubated with goat anti-mouse Alexa Fluor A488 (1/250) for 45 minutes before washing and treatment with 0.1% Sudan black B for 10 min to reduce autofluorescence. Slides were counterstained with ProLong® gold antifade fluorescent mounting media with DAPI (Molecular Probes). The reading was performed by microscope analysis at high power and then dual immunoflourescent staining was evaluated for validation.

### Staining Quantification

**Figure 1:**
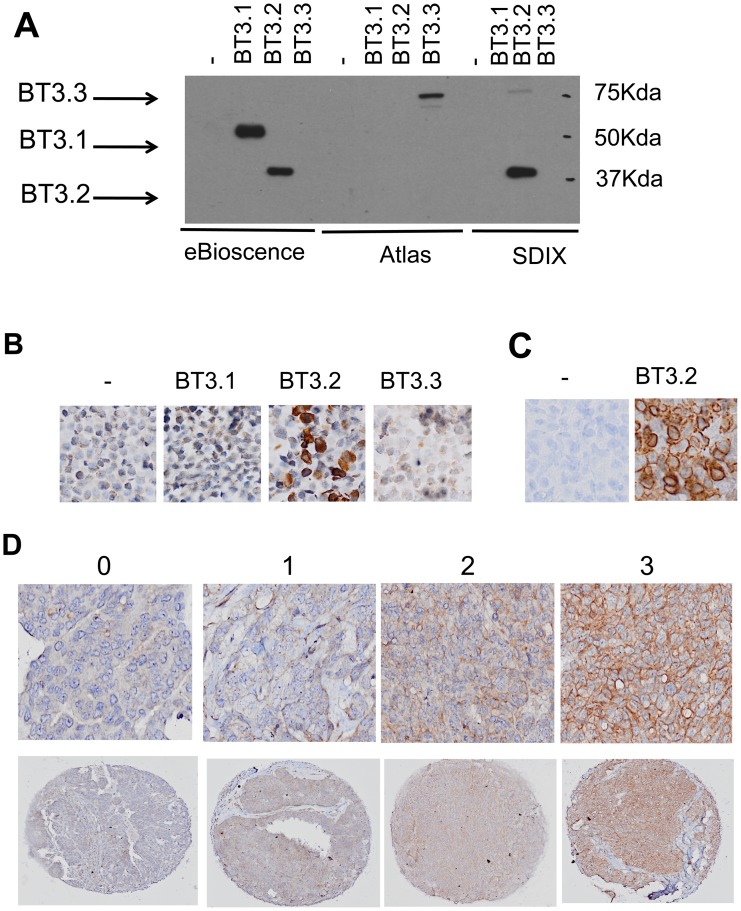
BT3.2 expression in ovarian cancer cells. **A**. Western-blot analysis of total protein extracts from TOV112D cell line infected with PLenti (vector control), BT3.1, BT3.2 or BT3.3 viral constructs. Extracts were loaded in triplicate on 10% SDS/PAGE gel and membranes were hybridized with anti-CD277 (eBioscience), anti-BT3.3 (Atlas Antibodies) and anti-BT3.2 (SDIX Inc.) as indicated at the bottom of the Figure. **B**. Immunohistochemistry analysis of paraffin-embedded cell pellets from TOV112D cell line infected with either PLenti (vector control), BT3.1, BT3.2 or the BT3.3 viral constructs. Only cells transfected with the BT3.2 construct stained positively with the anti-BT3.2 (SDIX Inc.). **C**. Immunohistochemistry of xenograft tumors from TOV112D transfected with either empty vector or BT3.2 construct. Only cells infected with the BT3.2 construct stained positively with the anti-BT3.2 (SDIX Inc.). **D**. Representative staining for immunohistochemistry of BT3.2 on a high-grade serous EOC TMA. From left to right: negative, low, moderate and high intensity.

Tumor sections were scanned and digitally visualized. For BT3.2, epithelial zones were scored according to the staining intensity of the epithelial membrane (value of 0 for absence, 1 for weak, 2 for moderate, 3 for high intensity) as illustrated in Figure 1. In cores where staining was of variable intensity the average intensity was reported. The quantification of infiltrating lymphocytes was carried out by counting the number of nucleated cells with positive staining in intraepithelial islets. Results were then categorized as 0 (no cells), 1 (0<n<10), 2 (10<n<90) and 3 (n>90) accordingly of the number of cells counted. The quantification of macrophages was carried out by evaluating the percentage of stained cells in the intraepithelial area. The relative density of CD206+cells was calculated by the ratio CD206+/CD68+ stained cells. Each array was independently analyzed in a blind study by two independent observers. Inter-rating correlation was >75%. When strong differences in scoring between the two observers (more than 1 unit per core) occurred the core was re-evaluated to reach a concordant scoring between the two observers. The average of all cores with cancer from the same patient was used for analysis.

### Western Blot Analysis

Cells were lysed with cold lysis buffer (10 mM Tris-HCl, pH 7.4, 150 mM NaCl, 1 mM EDTA, 1 mM DTT/1 mM NaF/0.5% NP-40/and protein inhibitors) and centrifuged for 10 min at 13000 rpm. Clear lysates were then boiled in loading buffer, separated by 10% SDS-PAGE, and transferred on a nitrocellulose membrane. Membranes were saturated with 5% milk/PBS/0.1% Tween 20. Immunodetection was done as described in the protocol of the ECL kit (Amersham Pharmacia): i.e. incubated 2 h at room temperature or O/N at 4°C with the specific antibody, washed with PBS/Tween 0.01% and incubated for another 30 min at room temperature with peroxidase conjugated antibodies (Santa-Cruz Biotechnology Inc.). Western-blot analysis was performed with beta-actin AC-15 (ab6276 from Abcam inc. MA, USA), anti-CD277/BT3.1 (#14–2779 from eBioscience, San Diego, CA, USA), anti-BT3.2 (SDIX, Newark DE, USA), anti-BT3.3 (HPA007904, Atlas Antibodies, Stockholm, Sweden). Experiments were done in triplicate.

### Statistical Analysis

The Pearson correlation test (two-tailed) was used to estimate the correlation with clinico-pathological variables and markers as continuous variables. Survival curves were plotted using the Kaplan-Meier curve analysis and the log-rank test was used to test for significant differences. Receiver operative characteristic (ROC) curves were used to determine the threshold value for each marker corresponding to the best sensitivity and specificity for patient progression before 18 months (early disease progression) or after 20 months (late disease progression) from initial diagnosis (p<0.05 and area>0.60) as already reported [5]. Univariate and multivariate Cox proportional hazard models were used to estimate the hazard ratio for each marker as continuous variables. Multivariate analysis was done using a forward stepwise hazard model on univariate analysis required for entry into the model. Only four variables were including in the multivariate Cox regression model to avoid over-fitting. Multivariate analysis was performed using an enter hazard model. Sample size (n >100 where n = 10k/p) and normal distribution of BT3.2 expression were considered before applying the Cox model. All statistical analyses were done using Statistical Package for the Social Sciences software version 11.0 (SPSS, Inc.), and statistical significance was set at *P*<0.05.

## Results

### Expression of BTN3A2/BT3.2 in EOC Tissues

To investigate the association between of BT3.2 expression in EOC cells and patient survival, we performed an immunohistochemistry analysis in a cohort of 199 high-grade serous ovarian cancer patients. First, we evaluated the specificity of available antibodies against BT3 isoforms. We found 3 different antibodies commercially available: anti-CD277 (eBioscience Inc.), anti-BT3.3 (Atlas) and anti-BT3.2 (from SDIX Inc.). Antibodies were tested by western-blotting on cellular extracts from the TOV112D ovarian cancer cell line infected with either BT3.1, or BT3.2 or BT3.3 retroviral particles. As seen in Figure 1A, the anti-CD277 recognized BT3.1 and BT3.2 isoforms. As expected, the anti-BT3.3 antibody exclusively recognized the BT3.3 isoform and the anti-BT3.2 specifically recognized the BT3.2 isoform. To confirm the specificity of the anti-BT3.2 antibody on paraffin embedded tissues, we also analyzed the staining of paraffin-embedded cell pellets and xenograft tissues from 112D TOV cells transfected with either an empty vector or BT3 isoform constructs. As seen in Figure 1B and 1C, the anti-BT3.2 antibody was able to stain only the BT3.2 cell pellet and BT3.2 xenograft tumor.

Membrane and cytoplasmic expression of BT3.2 in ovarian cancer tissues was observed and scored according to the intensity of staining as absent, low, moderate or strong (0, 1, 2, 3 respectively) (Figure 1D). Presence of BT3.2 protein was observed on epithelial cells and in some tissue cores, it could also be found in the stroma. Almost all EOC cores (n = 193, 97.5%) showed expression of BT3.2. On epithelial cells, both cytoplasmic and membrane staining were seen (Figure 1D) and varied from absent to strong (Figure 1D, Table 1). The epithelial BT3.2 expression levels did not significantly correlated with patient age at diagnosis or tumor grade. In contrast, patients with higher level of epithelial BT3.2 tend to have lower level of residual disease and lower stage disease (p = 0.058 and p = 0.043, respectively, Table 2).

**Table 2:** Pearson correlation test (two-tailed) between BT3.2 expression in EOC tissues and clinical parameters of patients.

		AGE	GRADE	STAGE	CA125	Res. Disease	Recurrence	DEATH
BT3.2	Correlation	−0.017	0.082	−0.145	0.071	−0.16	−0.218	−0.23
	p	0.812	0.249	0.043	0.391	0.058	0.004	0.001
	N	194	198	196	149	140	174	198

Table showing the coefficient of correlation with a Pearson correlation test (Correlation) between clinical parameters and BT3.2 staining intensity observed in the intra-epithelial area of ovarian tumor tissues. Grade and stage were evaluated according to the FIGO classification as described in Materials and Methods. Res. Disease is the amount of residual disease at primary resection of the tumor as classified in Table 1. Recurrence is the first event of ovarian cancer recurrence after the primary resection of the tumor. CA125 =  blood serum level CA125 at the date of primary resection of the ovarian tumor. P is the p value from Pearson correlation test. N is the number of cases included in the statistical analysis.

### BT3.2 Protein Expression is Associated with Overall Survival and Disease-free Progression

We have previously investigated the relation between BT3.2 mRNA expression and ovarian cancer patient prognosis. Kaplan-Meier and Cox proportional hazard model were used to estimate the association between BT3.2 mRNA and prognosis as defined by overall survival or disease-free survival (DFS) within 18 months after surgery [5]. In the present study, we investigated whether BT3.2 protein could also be of prognostic value. For this purpose, protein expression of BT3.2 was analyzed in 199 high-grade serous patients and statistical analyses were performed with respect to overall survival and DFS. The cohort characteristics are described in Table 1.

**Figure 2:**
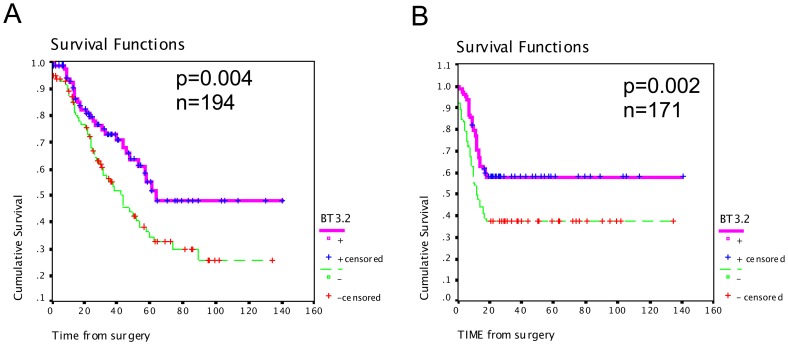
Kaplan Meier analysis of BT3.2 in high-grade serous EOC. Kaplan-Meier curves of overall survival (A) and disease-free survival (B) in 194 and 171 patients, respectively. Significance (p) is indicated by log rank.

Kaplan-Meier curves showed that there was a strong association between high expression of BT3.2 protein and increased overall patient survival (p = 0.014; log rank = 6.03) (Figure 2A). Mean survival interval was 85 months for patients with higher level of BT3.2 compared with 59 months for patients with low level of BT3.2. Similarly, patients with higher expression of BT3.2 had a mean progression interval of 86 months compared to 55 months for patients with low expression of BT3.2 (p = 0.002, log rank = 9.65) (Figure 2B).

In univariate Cox regression analysis, the continuous level of BT3.2 protein was evaluated to reflect the relation between increasing level of BT3.2 expression and improved prognosis. In this analysis, increased expression of BT3.2 was associated with a relatively high hazard risk (HR) for survival (HR = 0.651, 95%CI 0.479–0.884, p = 0.006, Table 3). Similarly, increased BT3.2 expression was significantly associated with later disease progression (HR = 0.642, %95CI 0.483–0.853, p = 0.002) (Table 3).

In multivariate Cox regression analysis, when standard prognostic variables were undertaken (age, stage and residual disease), BT3.2 remained an independent variable predicting a high risk of survival (HR = 0.597, %95CI 0.415–0.859, p = 0.005) and late progression risk in this multivariate model (HR = 0.675, %95CI 0.487–0.935, p = 0.018) (Table 3).

**Table 3:** Univariate and multivariate Cox regression analysis showing the statistical association between BT3.2 expression and outcome of EOC patients.

		Univariate analysis				Multivariate analysis			
		HR (95%CI)			p	HR (95%CI)			p
**OS**	AGE	0.996	(0.971	1.022)	0.781	0.996	(0.971	1.022)	0.781
	STAGE	2.17	(1.399	3.37)	0.001	1.145	(0.645	2.032)	0.643
	Res.Dis.	1.654	(1.323	2.068)	0	1.586	(1.233	2.04)	0
	BT3.2	0.651	(0.479	0.884)	0.006	0.597	(0.415	0.8590	0.005
**DFS**	AGE	0.998	(0.978	1.018)	0.832	0.997	(0.975	1.019)	0.765
	STAGE	1.796	(1.265	2.551)	0	1.353	(0.815	2.2460	0.243
	Res.Dis.	1.619	(1.320	1.987)	0	1.527	(1.214	1.922)	0
	BT3.2	0.642	90.483	0.853)	0.002	0.675	(0.487	0.935)	0.018

OS =  overall survival is the time of survival from the date of primary resection until event of death due to ovarian cancer. Res. Dis. =  amount of residual disease at time of primary resection of the ovarian tumor. Age =  age of patient at time of the first resection of the ovarian tumor. DFS =  disease free survival is the time from the first resection of the primary tumor until the first event of recurrence. HR =  hazard ratio. CI =  confidence interval. P =  p value.

### Relationship Between BT3.2 Expression and Immune Infiltrate

We hypothesized that the relation between epithelial BT3.2 expression and a good prognosis was due to the influence on tumor infiltrating immune cells, since it is known that B7 family molecules have co-regulatory functions on T cells. In addition, the level of intratumoral immune cell infiltration has been related to prognosis of ovarian cancer patients [31]. To investigate this hypothesis, we evaluated by immunohistochemistry the presence of T cells by CD3+, CD4+ and CD8+ staining in the same TMA used for BT3.2 analysis. We also assessed the importance of intratumoral B cell (CD20+) and macrophage (CD68+) infiltration. Two classes of macrophages have been described, anti-tumoral (M1) and pro-tumoral (M2). Since in most tumors, tumor-associated macrophages (TAM) have an M2-phenotype [32], [33], we determined the density of infiltrating CD206+ cells as a surrogate marker of alternative pro-tumoral M2 macrophages since they tend to express higher level of CD206 marker than M1 macrophages. To confirm the specificity of CD206 expression by macrophages in EOC tumors, we performed a control double immunofluorescent staining on a limited number of tissues (Figure S1). CD206 expression almost always co-localized with CD68 expression suggesting macrophage expression.

**Figure 3:**
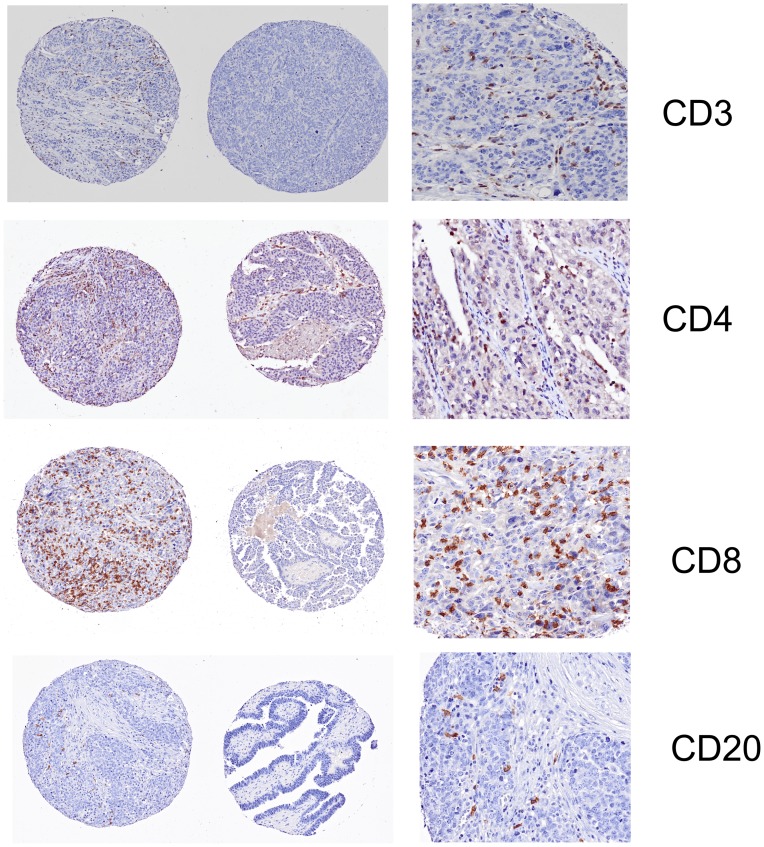
Representative staining for immunohistochemistry of tumor infiltrating lymphocytes. High and low density of CD3+ (T cells), CD4+ (T cells), CD8+ (T cells) and CD20+ (B cells) shown in left and middle panels. A magnification in the high infiltration area is shown on the right panel for each staining.

**Figure 4:**
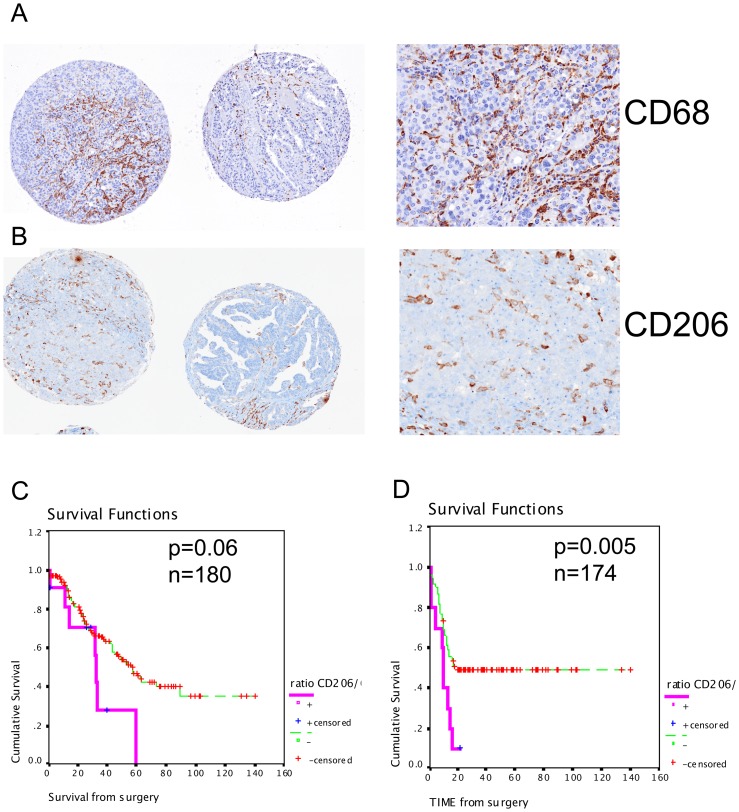
Representative staining for immunohistochemistry of infiltrating macrophages. High and low density of CD68+ (tumor-associated macrophages, TAM) (A) and CD206+ (M2 subtype of TAM) cells (B). Magnification in the high infiltration area is shown on the right panel for each staining (top). C and D. Kaplan-Meier analysis of the ratio of infiltrating intraepithelial CD206+/CD68+ cells representing the relative density of CD206+ M2 TAM over the total density of CD68+ intraepithelial infiltrating macrophages. Kaplan-Meier curves of overall survival (C) and disease-free survival (D) in 180 and 174 patients, respectively. Significance (p) is indicated by log rank.

As seen in Figure 3 and Figure 4A-B, immune infiltration of T cells, B cells and TAM was observed in EOC tissues at various densities in the intraepithelial areas. The intraepithelial infiltration of T cells, CD3+, CD4+ or CD8+, was highly correlated with the presence of CD20+ B cells (p<0.001) and CD68+ macrophages (P<0.001), including the CD206+ cells (Table 4). The intraepithelial infiltration of B cells was also associated with the presence of CD68+ and CD206+ cells (r = 0.364 and r = 0.171, p<0.001 and p = 0.022, respectively). Interestingly, the proportion of CD206+ cells relative to the total density of CD68+ macrophages, the ratio CD206+/CD68+ expression, was inversely correlated to the presence of CD3+T cells and tended to correlate with the presence of B cells (r = -0.216, p = 0.005 and r = -0.141, p = 0.063, respectively) (Table 4).

Significantly, the expression of BT3.2 by EOC cells was correlated with the intraepithelial infiltration of CD3+ cells (r = 0.174, p = 0.018), including CD8+ cells (r = 0.176, p = 0.018) and CD4+ cells (r = 0.272, p<0.001). No significant correlation between BT3.2 expression and the presence of CD20+ cells or macrophages was seen (p = 0.137, Table 4).

**Table 4:** Pearson correlation test (two-tailed) between BT3.2 expression and markers of intra-epithelial immune infiltrate in EOC tissues.

		*CD8*	*CD4*	*CD20*	*CD68*	*CD206*	*CD206/CD68*	*BT3.2*
CD3	C	0.663	0.657	0.309	0.555	0.436	−0.216	0.174
	p	0	0	0	0	0	0.005	0.018
	N	180	163	182	180	175	170	185
CD8	C	1	0.569	0.468	0.616	0.381	−0.161	0.176
	p	.	0	0	0	0	0.038	0.018
	N	181	159	178	175	171	166	180
CD4	C	0.569	1	0.29	0.56	0.495	−0.194	0.272
	p	0	.	0	0	0	0.013	0
	N	159	175	169	169	167	163	175
CD20	C	0.468	0.29	1	0.364	0.171	−0.141	0.108
	p	0	0	.	0	0.022	0.063	0.137
	N	178	169	192	185	179	174	191
CD68	C	0.616	0.56	0.364	1	0.468	−0.315	0.059
	p	0	0	0	.	0	0	0.42
	N	175	169	185	192	180	180	191
CD206	C	0.052	0.13	−0.068	0.381	0.495	0.171	0.128
	p	0.486	0.077	0.362	0	0	0.022	0.083
	N	181	185	183	171	167	179	184
CD206/CD68	C	−0.161	−0.194	−0.141	−0.315	0.173	1	−0.007
	p	0.038	0.013	0.063	0	0.02	.	0.353
	N	166	163	174	180	180	180	170

Table showing the Pearson coefficient of correlation C between staining observed for each marker in the intra-epithelial ovarian tumor tissues. Grade and stage were evaluated according to the FIGO classification. P is the p value from Pearson correlation test. N is the number of cases included in the statistical analysis for which the observed staining was reliable. CD206/CD68 =  ratio of CD206+/CD68+ staining in the intra-epithelial area of tumor. C =  Pearson correlation coefficient. P = p value.

### Association of Intratumoral Lymphocytes, Macrophages and EOC Patient Prognosis

As reported by others [31], the presence of CD4+ cells was also inversely correlated with early disease progression (r = -0.187, p = 0.021, Table 5). Kaplan-Meier curve analysis and log rank test confirmed that high intraepithelial density of CD4+ was associated with a better prognosis (Table S2). The mean progression interval of patients with high intra-epithelial CD4+ cells was 69 months, as compared to 59 months for patients with low intraepithelial CD4+ density (p = 0.011, log rank = 6.5). However, we did not observe any significant association between intraepithelial density of CD3+ or CD8+ cells and patient prognosis (Table 5 and Table S2). We found a significant association between CD20+ cell infiltration and overall survival (log rank p = 0.039), whereas only a trend toward a better disease-free survival was obtained (log Rank p = 0.0677). However, the association with overall survival was not seen when CD20+ was considered as a continuous variable in univariate Cox regression model (p = 0.21).

Whilst we did not observe a correlation between the density of CD68+ or CD206+ cells and patient prognosis (Table 5), an increased ratio of CD206+ relative to CD68+ cells was positively correlated with disease progression (r = 0.157, p = 0.049, Table 5). This relation was confirmed by Kaplan-Meier curve analysis (p = 0.005, log rank = 7.83, Figure 4D, Table S2) and univariate Cox regression analysis (HR = 1.355, 95%CI 1.223–5.332, p = 0.044). Furthermore, the CD206+ relative density tended to associate with poor overall survival (log rank = 3.53, p = 0.06) (Figure 4C, Table S2) with a hazard ratio of 2.077 (%CI 95, 0.947–4.558, p = 0.068).

**Table 5:** Pearson correlation test (two-tailed) between intra-epithelial immune infiltrate and clinical parameters in EOC patients.

		*Age*	*Grade*	*Stage*	*Recurrence*	*Death*
CD3	C	0.033	0.105	−0.21	−0.109	−0.133
	p	0.655	0.152	0.004	0.168	0.071
	N	182	186	184	163	186
CD8	C	0.023	0.133	−0.156	−0.096	−0.04
	p	0.763	0.073	0.037	0.23	0.595
	N	177	181	180	160	181
CD4	C	−0.013	0.163	−0.2	−0.187	−0.051
	p	0.87	0.031	0.008	0.021	0.505
	N	171	175	173	153	175
CD20	C	−0.039	0.015	−0.075	−0.136	−0.089
	p	0.596	0.833	0.301	0.079	0.221
	N	188	192	190	168	192
CD68	C	0.131	0.066	−0.168	−0.019	0.057
	p	0.072	0.363	0.02	0.809	0.429
	N	189	192	190	170	192
CD206	C	0.062	0.139	−0.075	0.03	0.06
	p	0.41	0.06	0.31	0.709	0.415
	N	181	185	183	161	185

Table showing the coefficient of correlation with a Pearson correlation test (Pearson Correlation) between clinical parameters and immune infiltrate density in the intra-epithelial ovarian tumor tissues. Grade and stage were evaluated according to the FIGO classification. Res. Dis. =  amount of residual disease at primary resection of the tumor as described in Table 1. Recurrence is the first event of ovarian cancer recurrence after the primary resection of the tumor. Death is the event of death due to ovarian cancer. CA125 =  blood serum level CA125 at the date of primary resection of the ovarian tumor. P is the p value from Pearson correlation test. N is the number of cases included in the statistical analysis. C =  Pearson correlation coefficient. P = p value. N =  number of cases.

## Discussion

In this study, immunohistochemistry revealed BTN3A2/BT3.2 staining in high-grade EOC serous tumors from 199 patients. Expression was detected at high frequency (97.5%) in accordance with previous data analyzing the mRNA expression in serous EOC tissues [5]. The important aspect of this study is the confirmation of BT3.2 as a potential prognostic marker for high-grade serous EOC at the protein level. The initial study identifying BT3.2 mRNA as a prognostic marker was limited to a relatively small set of 52 cases. Here, not only we confirmed the relation between BT3.2 and a better outcome in a large cohort, but also at protein level by immunohistochemistry. This constitutes a more easily transposable technique in a clinical pathology department than mRNA detection such as Q-PCR. Patients with high epithelial staining of BT3.2 had 1.53 times less risk of dying from the disease than patients with low level of BT3.2 expression. The association of BT3.2 to survival and to disease progression was an independent parameter within a multivariate Cox-regression analysis (Table 3). Combination with other independent molecular markers could improve its clinical performance as to date no individual marker has demonstrated sufficient sensitivity and specificity.

A limitation of our study is the low number of patients with no residual disease (n = 23), which did not allow us to analyze them as an independent cohort. Optimally debulked patients, overall have better prognosis than patients with residual disease [34] for which a prognostic biomarker may be less informative. However, 84% of the cohort analyzed here had residual disease (Table 1). Notably, 53% of patients showed a disease progression within 18 months following ovariectomy and 41% died from the disease. Thereby, even a cohort of patients with residual disease would benefit from prognostic biomarkers to aid patient management. In addition, the presence of residual disease is in part due to a surgical limitation rather than the biological behavior of the tumor, so it is reasonable to speculate than BT3.2 will also be a significant prognosis marker in a cohort of optimally debulked patients. This hypothesis still needs to be validated in such a cohort of EOC patients. Knowing that immune regulation is an important factor in the outcome of several epithelial cancers, it is possible than BT3.2 has a similar role in other carcinomas. In line with this hypothesis we also observed expression of BT3.2 in breast, renal and colon cancer tissues (Figure S2). However, the association of this expression and the outcome of patients for these cancers should be specifically investigated to confirm this hypothesis.

Ovarian cancers, as do most cancers, involved multimolecular events, which regulate the mechanisms of cancer progression and antitumoral response. In the last decade, a number of studies have shown a major role for immune cells in the host anti-tumor response to ovarian cancer [31] and tumor-mediated immune evasion. The first reported observation [35] found that CD3^+^ T cells were associated with better survial and disease-free-progression in a cohort containing several subtypes of EOC. While some studies confirmed the relation between intratumoral CD3+T cells density and EOC patient prognosis [36], [37], [38], others did not [39], [40] [31], [37], [41]. Together, these observations suggest that complex regulatory factors govern tumoral microenvironement and immune function. To better understand the mechanism and tumor factors involved in T cell infiltration of ovarian tumors, several groups have investigated the presence of intratumoral subtypes of T cells, macrophages (TAM), B cells and immune regulatory factors [42], levels of cytokines such as TNF or IFNγ [31]. However, mechanisms involved in the immune surveillance of ovarian cancer and the role of specific immune cells are not yet fully understood.

Recently, new members of the B7 family, the butyrophilins, attracted a strong interest as new T cell regulatory molecules [43]. First, BTNL2 was described as a negative T cell costimulatory molecule in a murine *in vitro* assay and in a murine intestinal inflammation model [17]–[18]. The murine BT1.1 and BT2.2 were also identified as co-inhibitors of T cell activation [19]. It is unknown whether human butyrophilin molecules share similar immune regulating functions. In human, BT3 is expressed in various immune cells and tumor cell lines including EOC cells [23]. A recent study also reported negative co-stimulatory functions for BT3.3 [22], but not BT3.1, on human blood T cells using antibodies against the BT3.3 isoform. Conversely, another study proposed that CD277 (BT3) inhibited human T cells proliferation when expressed by APC [23]. A last study showed that CD277 engagment stimulated the activation of human T cells *in vitro* [24]. More interestingly, in this study, a differential role of the BT3 isoforms was reported [24] where BT3.2 was the main isoform expressed by NK cells and the only one able to inhibit NK-induced cytokine production. Altogether, these results suggest that the role of the BT3 isoforms is complex, specific to cell type and regulated by the combination of isoforms. This also raised the question whether BT3 isoforms share the same receptor or whether the 3 isoforms bind to specific receptors, which could transmit different and perhaps opposite activation signals. In addition, one could also speculate that CD277 expressed by myeloid APCs (potentially pro-inflammatory as a ligand) could have a different role than CD277 expressed by T cells (potentially anti-inflammatory as a receptor) or by epithelial cells. We do not know yet if BT3 isoforms expressed by EOC cells have similar functions than isoforms expressed by immune cells. Further observations are needed to understand the exact role of BT3 isoforms in a context specific manner.

From previous studies reporting an immunosuppressive role of BT3 when expressed by immune cells, one could speculate that in cancer, BT3.2 would favor the tumoral “immune escape” by decreasing T cell activation. In this context, high expression of BT3.2 would be related to worse patient prognosis. However, it was also reported that factors, such as IFN-γ and TNF, known to stimulate the anti-tumoral response, can also up-regulate the expression of BT3 molecules [12]. The analysis presented here reveals an interesting profile of BT3.2 by EOC cells (Figure 1 and Figure 2). We found that almost all EOC cells expressed BT3.2, but at different levels. A higher expression of BT3.2 by these cells is strongly associated not only with better patient prognosis, but also with a higher density of T cells, particularly CD4+ cells. Often used as markers of T lymphocytes, CD4 and CD8 are generally associated with T cells despite their low expression being reported in other cell types. The latter result is in line with recent observations, where the presence of ovarian tumor-infiltrating T-cells was associated with a longer patient survival and a longer progression interval (Table 4). Altogether, these results suggest that BT3.2 expression by EOC cells may regulate the tumor immune microenvironment and favor an anti-tumoral immune response. To confirm this hypothesis, further studies are needed to explore the co-stimulatory functions of BT3 and their role in regulating the tumoral immune milieu.

Another interesting observation revealed by this study is the relation between the TAM and EOC patient prognosis. A few immunohistochemistry studies have reported a lack of association between TAM and outcome in ovarian cancer tissues [38], [40], [44]. However, these studies did not distinguish between polarized M1 or M2 macrophages, since general antibodies against CD68 or CD36 were used. M1 macrophages, also named classically activated macrophages, are potent effectors of Th1 responses and producers of pro-inflammatory cytokines. They are thought to participate in the immune tumor resistance [45]. In contrast, M2 macrophages, also known as alternative macrophages, have a crucial role to limit the anti-tumoral immune response and are thought to foster immune tolerance. M1 and M2 macrophages are distinguishable by the distinct cytokines and membrane receptors they express. CD206 is generally highly expressed by M2 polarized macrophages and considerably less by M1 macrophages, so it is commonly used to identified M2 macrophages [32], [45]. Despite the lack of association between the density of CD206+ cells and outcome in EOC patients in our study, we found that a higher proportion of CD206+ cells relative to the total amount of TAM (CD68+) was strongly associated with early disease progression (Figure 4C-D). This is in line with previous reports showing that *in vitro* M2 tumor macrophages were poorly cytotoxic against neoplasic cells and produced growth factors for cancer cells [46]. Only a few studies have described the phenotype of TAM in ovarian cancer tissues. As we observed in our study, the majority of TAM in cancerous tissues is a M2 CD206+ population [32], [33], [47], [48], [49]. Here, we show for the first time that the relative density of intraepithelial CD206+/CD68+ cells reflecting M2 TAM, more than the overall quantity of TAM, is a critical factor associated with a shorter progression-free interval. However, this observation needs to be validated in an independent cohort.

In summary, we confirmed that BTN3A2/BT3.2 is a new promising prognostic marker for patients with high grade serous EOC. As for most biomarkers, it will need to be validated in an independent cohort of patients to confirm its clinical utility. Even if the biological function of BT3.2 still remains to be determined, our data suggest that epithelial expression of BT3.2 is associated with a beneficial infiltration of immune cells, particularly CD4+ T cells. This has an important implication for understanding the biology of these BT3 molecules in the regulation of the anti-tumoral immune response and EOC patient management after surgery. Further “*in vitro”* and “*in vivo*” experiments need to be done to better understand the mechanism involved in the immune regulation by epithelial BT3 in cancer.

## Supporting Information

Figure S1 **Expression of CD206+ cells by CD68+ macrophages.** Representative image. Blue DAPI signal in nuclei. Image 20X. **A**. Macrophage CD68+ staining (red). **B.** CD206+ staining (green). **C.** Merge staining showing the colocalisation of CD68 and CD206 markers (orange). CD206 expression is almost exclusively co-localizing with CD68 staining.
(TIF)

Figure S2 **Expression of BT3.2 by other epithelial cancers.** Strong to moderate staining of BT3.2 observed in colon, renal and breast cancer tissues. Weak staining observed in prostate cancer tissue.
(TIF)

Table S1 **Primary antibodies and conditions used for immunohistochemistry on tissue microarray.**
(DOCX)

Table S2 **Kaplan-Meier analysis of immune infiltrate in 199 EOC patients.** NA: Not applicable when p value >0.05. Mean in months from the first resection of the ovarian tumor until an event of recurrence (disease free survival) or death (overall survival). Bold indicates significant p values<0.05.
(DOCX)
