# Supplementary figures and images for: BTN3A2 Expression in Epithelial Ovarian Cancer Is Associated with Higher Tumor Infiltrating T Cells and a Better Prognosis
Source: PLoS One. 2012 Jun 7;7(6):e38541. doi: 10.1371/journal.pone.0038541 (PMC3369854; doi:10.1371/journal.pone.0038541)

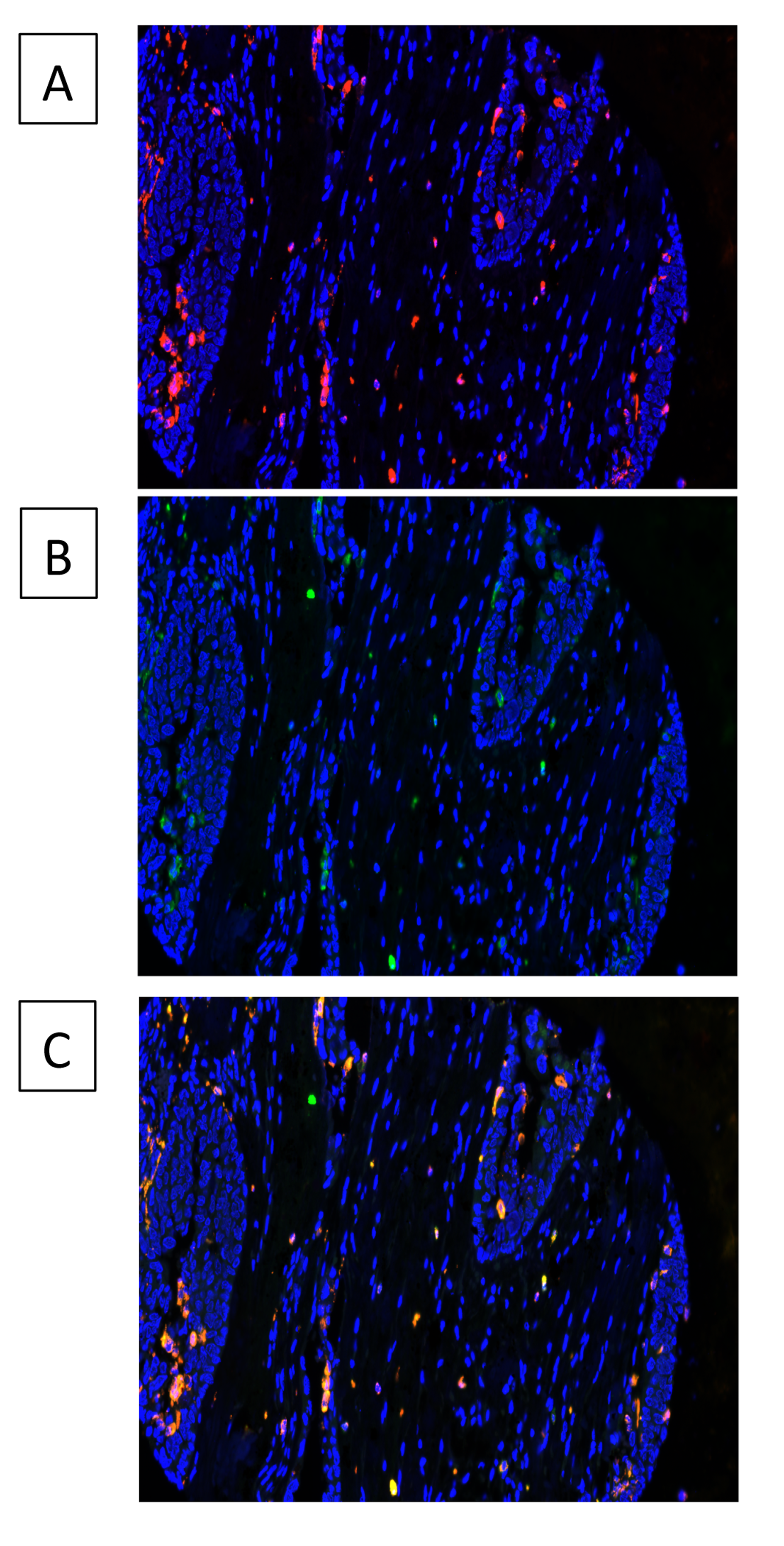

Supplement: Figure S1 — Expression of CD206+ cells by CD68+ macrophages. Representative image. Blue DAPI signal in nuclei. Image 20X. A. Macrophage CD68+ staining (red). B. CD206+ staining (green). C. Merge staining showing the colocalisation of CD68 and CD206 markers (orange). CD206 expression is almost exclusively co-localizing with CD68 staining. (TIF) [file pone.0038541.s001.tif]

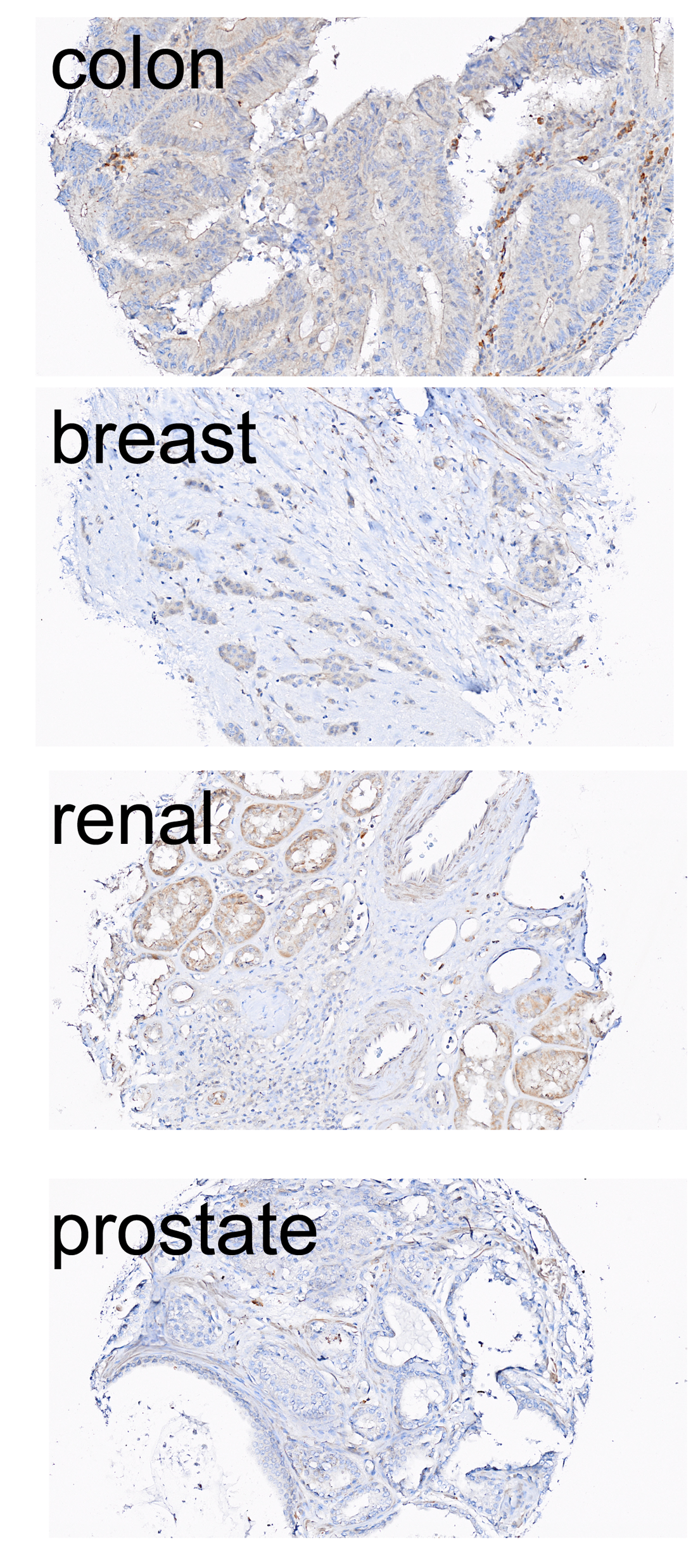

Supplement: Figure S2 — Expression of BT3.2 by other epithelial cancers. Strong to moderate staining of BT3.2 observed in colon, renal and breast cancer tissues. Weak staining observed in prostate cancer tissue. (TIF) [file pone.0038541.s002.tif]
