# Supplementary material for: BTN3A2 Expression in Epithelial Ovarian Cancer Is Associated with Higher Tumor Infiltrating T Cells and a Better Prognosis
Source: PLoS One. 2012 Jun 7;7(6):e38541. doi: 10.1371/journal.pone.0038541 (PMC3369854; doi:10.1371/journal.pone.0038541)
Supplement: Table S1 — Primary antibodies and conditions used for immunohistochemistry on tissue microarray. (DOCX) [file pone.0038541.s003.docx]

**Table S1: Primary antibodies and conditions used for immunohistochemistry on tissue microarray.**

| **Antibody** | **Source** | **Method** | **H_2_O_2_ treatment** | **Antigen retrieval Condition** | **Dilution**  **(staining time)** |
| --- | --- | --- | --- | --- | --- |
|  |  |  |  |  |  |
| CD3 (clone PS1) | Novocastra | immunoperoxidase | 0.60% | Tris-ETDA | 1/50 (60min) |
| CD4 (clone IF6) |  | Ventana | Ultraview Dab kit |  |  |
| CD8 (clone C8/144B) | Dako | immunoperoxidase | 0.60% | Tris-ETDA | 1/25 (60min) |
| CD20cy (clone L26) | Dako | immunoperoxidase | 0.60% | Tris-ETDA | 1/200 (60min) |
| CD68 (clone PG-M1) | Dako | immunoperoxidase | 0.60% | Tris-ETDA | 1/50 (60min) |
| CD206 (clone 5C11) | Abnova | Ventana | Ultraview Dab kit | Citrate | 1/500 (60 min) |
| BT3.2 | SDIX | immunoperoxidase | 3% | Tris-ETDA | 1/100 (120min) |
