# Supplementary material for: BTN3A2 Expression in Epithelial Ovarian Cancer Is Associated with Higher Tumor Infiltrating T Cells and a Better Prognosis
Source: PLoS One. 2012 Jun 7;7(6):e38541. doi: 10.1371/journal.pone.0038541 (PMC3369854; doi:10.1371/journal.pone.0038541)
Supplement: Table S2 — Kaplan-Meier analysis of immune infiltrate in 199 EOC patients. NA: Not applicable when p value >0.05. Mean in months from the first resection of the ovarian tumor until an event of recurrence (disease free survival) or death (overall survival). Bold indicates significant p values<0.05. (DOCX) [file pone.0038541.s004.docx]

**Table S2: Kaplan-Meier analysis of immune infiltrate in 199 EOC patients.**

|  | **overall survival** | | | **disease free survival** | | |
| --- | --- | --- | --- | --- | --- | --- |
| **Marker** | p | log rank | mean months | p | log rank | mean months |
| CD3 | 0.176 | NA | NA | 0.418 | NA | NA |
| CD4 | 0.134 | NA | NA | **0.011** | 6.5 | 69-59 |
| CD8 | 0.199 | NA | NA | 0.714 | NA | NA |
| CD20 | 0.039 | NA | NA | 0.067 | NA | NA |
| CD68 | 0.662 | NA | NA | 0.391 | NA | NA |
| CD206 | 0.457 | NA | NA | 0.075 | NA | NA |
| CD206/CD68 | **0.06** | NA | NA | **0.005** | 7.83 | 10-78 |

NA: not applicable when p value >0.05. Mean in months from the first resection of the ovarian tumor until an event of recurrence (disease free survival) or death (overall survival). Bold indicates significant p values<0.05.
